# The differential demographic pattern of coronavirus disease 2019 fatality outside Hubei and from six hospitals in Hubei, China: a descriptive analysis

**DOI:** 10.1186/s12879-021-06187-4

**Published:** 2021-05-26

**Authors:** Qing-Bin Lu, Hai-Yang Zhang, Tian-Le Che, Han Zhao, Xi Chen, Rui Li, Wan-Li Jiang, Hao-Long Zeng, Xiao-Ai Zhang, Hui Long, Qiang Wang, Ming-Qing Wu, Michael P. Ward, Yue Chen, Zhi-Jie Zhang, Yang Yang, Li-Qun Fang, Wei Liu

**Affiliations:** 1 Department of Laboratorial Science and Technology, School of Public Health, Peking University, Beijing, 100191 P. R. China; 2 State Key Laboratory of Pathogen and Biosecurity, Beijing Institute of Microbiology and Epidemiology, Beijing, 100071 P. R. China; 3 School of Mathematical Sciences, Beijing Normal University, Beijing, 100875 P.R. China; 4 Department of Thoracic and Vascular Surgery, Wuhan No. 1 Hospital, Tongji Medical College, Huazhong University of Science and Technology, Wuhan, 430022 P. R. China; 5 Department of Healthcare Management, School of Health Sciences, Wuhan University, Wuhan, 430071 P. R. China; 6 Global Health Institute, Wuhan University, Wuhan, 430072 P. R. China; 7 Department of Thoracic Surgery, Renmin Hospital of Wuhan University, Wuhan, 430060 P. R. China; 8 Department of Laboratory Medicine, Tongji Hospital, Tongji Medical College, Huazhong University of Science and Technology, Wuhan, 430030 China; 9 Tianyou Hospital affiliated to Wuhan University of Science and Technology, Wuhan, 430064 P. R. China; 10 Institute of Infection, Immunology and Tumor Microenvironent, Hubei Province Key Laboratory of Occupational Hazard Identification and Control, Medical College, Wuhan University of Science and Technology, Wuhan, 430065 P. R. China; 11 Sydney School of Veterinary Science, The University of Sydney, Camden, NSW Australia; 12 Department of Epidemiology and Community Medicine, Faculty of Medicine, University of Ottawa, 451 Smyth Rd, Ottawa, Ontario Canada; 13 Department of Epidemiology and Health Statistics, School of Public Health, Fudan University, Shanghai, P. R. China; 14 Department of Biostatistics, College of Public Health and Health Professions, and Emerging Pathogens Institute, University of Florida, Gainesville, Florida, USA

**Keywords:** COVID-19, Case fatality rate, China

## Abstract

**Background:**

The coronavirus disease 2019 (COVID-19) epidemic has been largely controlled in China, to the point where case fatality rate (CFR) data can be comprehensively evaluated.

**Methods:**

Data on confirmed patients, with a final outcome reported as of 29 March 2020, were obtained from official websites and other internet sources. The hospitalized CFR (HCFR) was estimated, epidemiological features described, and risk factors for a fatal outcome identified.

**Results:**

The overall HCFR in China was estimated to be 4.6% (95% CI 4.5–4.8%, *P* < 0.001). It increased with age and was higher in males than females. Although the highest HCFR observed was in male patients ≥70 years old, the relative risks for death outcome by sex varied across age groups, and the greatest HCFR risk ratio for males vs. females was shown in the age group of 50–60 years, higher than age groups of 60–70 and ≥ 70 years. Differential age/sex HCFR patterns across geographical regions were found: the age effect on HCFR was greater in other provinces outside Hubei than in Wuhan. An effect of longer interval from symptom onset to admission was only observed outside Hubei, not in Wuhan. By performing multivariate analysis and survival analysis, the higher HCFR was associated with older age (both *P* < 0.001), and male sex (both *P* < 0.001). Only in regions outside Hubei, longer interval from symptom onset to admission, were associated with higher HCFR.

**Conclusions:**

This up-to-date and comprehensive picture of COVID-19 HCFR and its drivers will help healthcare givers target limited medical resources to patients with high risk of fatality.

**Supplementary Information:**

The online version contains supplementary material available at 10.1186/s12879-021-06187-4.

## Background

Coronavirus disease 2019 (COVID-19) was first reported in Wuhan, central China in early December 2019 and rapidly swept through the whole country during the following months. The disease was commonly manifested as influenza-like illness including fever, cough, myalgia, sore throat and fatigue [1], but severe pneumonia developed in some patients who can experience acute respiratory distress syndrome and die of septic shock and multi-organ failure [2–6]. As of 2 February 2021, more than 102 million cases had been reported worldwide, with more than 2.2 million deaths from 216 countries. Case fatality rate (CFR) is a key epidemiologic parameter, which appears to vary substantially by geographic region such as high CFRs in Yemen, Mexico, Sudan, Italy and low CFRs in Qatar, Singapore and Malaysia. The estimation of CFR remains challenging, especially in the case of countries in which the epidemic is continuing [7]. CFR estimation is based on dividing the observed number of fatalities by the number of confirmed cases. However, such an estimate might be severely biased due to both the long time from hospitalization to death or recovery, and the underreporting of mild or asymptomatic infections, especially during the early phase of the epidemic [3, 8–10].

Most of the previously published CFR data in China were derived from the first 2 month of documented COVID-19 cases, which do not reflect the entire epidemic situation, and should be inevitably biased due to the uncertainty of the final patient outcome [10–12]. Now the epidemic has been largely brought to an end, with less than 10 new cases reported daily in China. This presents an opportunity to gain an accurate estimation of the mortality data in China and to identify populations at high risk for COVID-19 related deaths, to support the development and implementation of effective public health surveillance and mitigation efforts for the current COVID-19 global pandemic. Here, by using a most updated national dataset from Wuhan and provinces outside Hubei, we aimed to describe the key characteristics of deceased COVID-19 patients in China, to infer the different features from Wuhan and provinces outside Hubei, and to examine the epidemiological pattern of COVID-19 related death that might differ between age, sex, and epidemic regions and throughout the entire epidemic process.

## Methods

### Data sources

To obtain sufficient epidemic information of COVID-19 cases for analysis, we created our COVID-19 cases database from multiple sources [13]. First, we collected data from website of official government [14] and peer-reviewed papers [15]. Second, the social media accounts and news websites were searched for identifying the basic demographic characteristics (age, sex, and city) and other vital information (starting and ending dates of probable exposure, date of symptom onset, laboratory diagnosis status and associated epidemiological cluster) of COVID-19 cases [16]. Third, the individual case data for the estimate of hospitalized case fatality rate (HCFR) in Wuhan City were from six hospitals in Wuhan (Wuhan No. 1 Hospital, Renmin Hospital of Wuhan University, Tongji Hospital affiliated to Huazhong University of Science and Technology, Tianyou Hospital affiliated to Wuhan University of Science and Technology, Puren Hospital affiliated to Wuhan University of Science and Technology and Huarun Wugang General Hospital). All data were double-input and cross-validated by our trained staffs. This study was approved by the institutional review board of the Beijing Institute of Microbiology and Epidemiology (Beijing, China). All identifiable personal information was removed from the data by the six hospitals in Wuhan before any analysis.

All the patients were admitted, confirmed, treated and discharged according to the clinical criteria of diagnosis and discharge standards for “Diagnosis and Treatment Scheme of New Coronavirus Infected Pneumonia” [17]. Briefly, the patients who had epidemiology history, clinical manifestations that mimic COVID-19 were diagnosed after examination of SARS-CoV-2 RNA by real time polymerase chain reaction (RT-PCR) with/without chest computed tomography scanning.

Based on the three data sources, the number of all confirmed patients with clinical outcomes (death or recovered) in all provinces across the mainland of China as of 29 March 2020 were compiled at the city level and were used in the analysis to describe the epidemiology of COVID-19 related mortality in the mainland of China. Data on individual case were collected including all death patients and a part of survival patients outside Hubei province as of 29 March 2020, and all patients from the six hospitals in Wuhan City, of which the information of basic demographic information, clinical severity at diagnosis (severe vs. mild), and dates of symptom onset, diagnosis, hospitalization, and discharge or death were collected, which were used to analyze age- and sex- specific HCFRs and compare their difference between Wuhan and outside Hubei. All the information was double-entered by the researchers. The patients were categorized as either mild or severe disease as defined by National Health Commission of the People’s Republic of China [18]. Population data at the prefecture (city) level in 2017 were obtained from the National Bureau of Statistics of the People’s Republic of China.

### Statistical analysis

The HCFR were calculated based on the hospitalized patients in this study. Data on individual case with the information of age, sex, the interval from symptom onset to admission and clinical outcome were used to analyze the risk factor for fatal outcome, among those who had dates from symptom onset to death/discharge of hospitals were used to analyze survival probability over time for fatal cases and related factors for clinical course. Categorical variables were compared between groups with χ^2^ or Fisher’s exact tests, and the Kruskal-Wallis test and Wilcoxon rank sum test were conducted to compare the differences for more than two groups and two groups of continuous variables with abnormal distribution, respectively. A logistic regression model was used to identify risk factors for fatal outcome, using a case-control study. Ordinal Logistic regression model was used to analyze the related factors with clinical course of death cases. For both models, the considered variables included age, sex and interval from symptom onset to admission. The competing risk model was presented to identify the potential risk factors for death considering discharge as the competing event [19]. The nonparametric cumulative incidence curve was plotted. Sub-distribution hazard ratio (SHR) of different groups by age, sex and the interval from symptom onset to admission on death and 95% confidence interval (CI) were estimated. All statistical tests were 2-tailed at a nominal significance level of 0.05. All analyses were performed using Stata 14.0 (Stata Corp LP, College Station, TX).

## Results

### Epidemiological description of COVID-19 related mortality

Based on public released national data, a total of 3769 SARS-COV-2 related deaths were identified among 81,470 confirmed cases up until 29 May 2020, an overall HCFR was estimated to be 4.6% (95% CI 4.5–4.8%, *P* < 0.001). Among the 3769 death cases, 2428 (64.4%) were male and 3089 (82.0%) were 60 years of age or older. The median age of all deaths was 70 (IQR 63–78) years.

Fatal cases were reported from 26 provinces, with the highest HCFR reported from Hubei Province (97.1%, 3651/3769), particularly from Wuhan (79.9%, 3011/3769), followed by Xiaogan city (3.4%, 129/3769), Huanggang city (3.3%, 125/3769), and Ezhou city (1.6%, 59/3769) in Hubei Province (Fig. S1; Table S1). Provinces outside Hubei collectively reported 2.9% (118/3769) of all fatal in the country, yielding a HCFR of 0.9, 95% CI (0.7–1.0%), significantly lower than that of Hubei Province (5.4, 95% CI 5.2–5.6%, 3651/67801, *P* < 0.001, Fig. S1; Table S1). The highest HCFR outside Hubei was recorded in Xinjiang Uygur Autonomous Region (4.0, 95%CI 0.8–11.1%), followed by Hainan Province (3.6, 95% CI 1.3–7.6%) and Heilongjiang Province (2.7, 95% CI 1.4–4.5%). No deaths were reported in Jiangsu, Shanxi, Ningxia, Qinghai or Tibet provinces (autonomous region). The mortality rate in Wuhan city was 28 (95% CI 27–29) per 100,000, higher than other cities in Hubei Province (*P* < 0.001). Within Wuhan city, the highest HCFR was observed in Qingshan District (8.3, 95% CI 7.3–9.4%, 225/2964), while the highest mortality rate was observed in Hannan District [94 (95%CI 79–112) per 100,000].

Detailed information was obtained from all the 118 fatal patients outside Hubei Province, among whom 63.0% were male and the median age was 73 (65–80) years, older than that of all fatal cases nationwide.

For comparison, we used 443 fatal cases selected from six designated hospitals in Wuhan, with a comparable age (73 [IQR 64–81] years) and sex (61.6% male) distribution as those of fatal patients outside Hubei Province (Table 1).

**Table 1:** Epidemiological and clinical characteristics of the 561 fatal cases with SARS-COV-2 infection as of 29 March 2020, the mainland of China

Characteristic	All fatal cases (n=561)	Outside Hubei (n=118)	Hospitals in Wuhan (n=443)	***P*** value
Age, years, median (IQR)	73 (64–81)	73 (65–80)	73 (64–81)	0.586
<10	0 (0)	0 (0)	0 (0)	0.373
10–	0 (0)	0 (0)	0 (0)	
20–	1 (0.18)	1 (0.85)	0 (0)	
30–	10 (1.78)	3 (2.54)	7 (1.58)	
40–	12 (2.14)	1 (0.85)	11 (2.48)	
50–	61 (10.87)	12 (10.17)	49 (11.06)	
60–	144 (25.67)	30 (25.42)	114 (25.73)	
70–	161 (28.70)	38 (32.20)	123 (27.77)	
≥80	172 (30.66)	33 (27.97)	139 (31.38)	
Sex, n (%)				0.829
Female	214 (38.15)	44 (37.29)	170 (38.37)	
Male	347 (61.85)	74 (62.71)	273 (61.63)	
Interval from disease onset to diagnosis, days, median (IQR)	8 (3–14)	6 (2–9)	10 (4–15)	<0.001
1–5	168 (29.95)	46 (38.98)	122 (27.54)	<0.001
6–10	157 (27.99)	47 (39.83)	110 (24.83)	
>10	236 (42.07)	25 (21.19)	211 (47.63)	
Interval from disease onset to admission, days, median (IQR)	6 (2–11)	6 (2–8)	6 (2–11)	0.054
1–5	255 (45.45)	59 (50.00)	196 (44.24)	0.003
6–10	137 (24.42)	38 (32.20)	99 (22.35)	
>10	169 (30.12)	21 (17.80)	148 (33.41)	
Severity of disease				<0.001
Mild	222 (39.57)	6 (5.08)	216 (48.76)	
Severe	339 (60.43)	112 (94.92)	227 (51.24)	
Clinical course, median (IQR)	15 (9–22)	17 (10–25)	14 (9–22)	0.028
Mild	15 (9–23)	17 (5–32)	15 (9–22)	0.847
Severe	15 (9–22)	17 (10–25)	14 (9–21)	0.025

*IQR* Interquartile range.

For both groups, the HCFR increased dramatically with age with a highly similar trend, i.e., starting from no deaths under 20 years of age, to a very low HCFR < 40 years of age, steadily increasing to > 10% in 50–60 years old, and then higher in those ≥60 years of age (Table 1). Longer interval from symptom onset to diagnosis and higher proportion of the interval > 10 days from symptom onset to admission were observed for fatal patients from Wuhan than those from outside Hubei (10 vs. 6 days, *P* < 0.001 and 33.4% vs. 17.8%, *P* = 0.003, respectively). It is noteworthy that a significantly higher proportion of fatal cases were entered hospital with mild disease in Wuhan than outside Hubei Province (48.8% vs. 5.1%, *P* < 0.001), which was also related to a shorter clinical course (14 [IQR 9–22] vs. 17 [IQR 10–25] days, *P* = 0.028).

### Age- and sex-specific HCFR that differed between Wuhan and outside Hubei

**Fig. 1:**
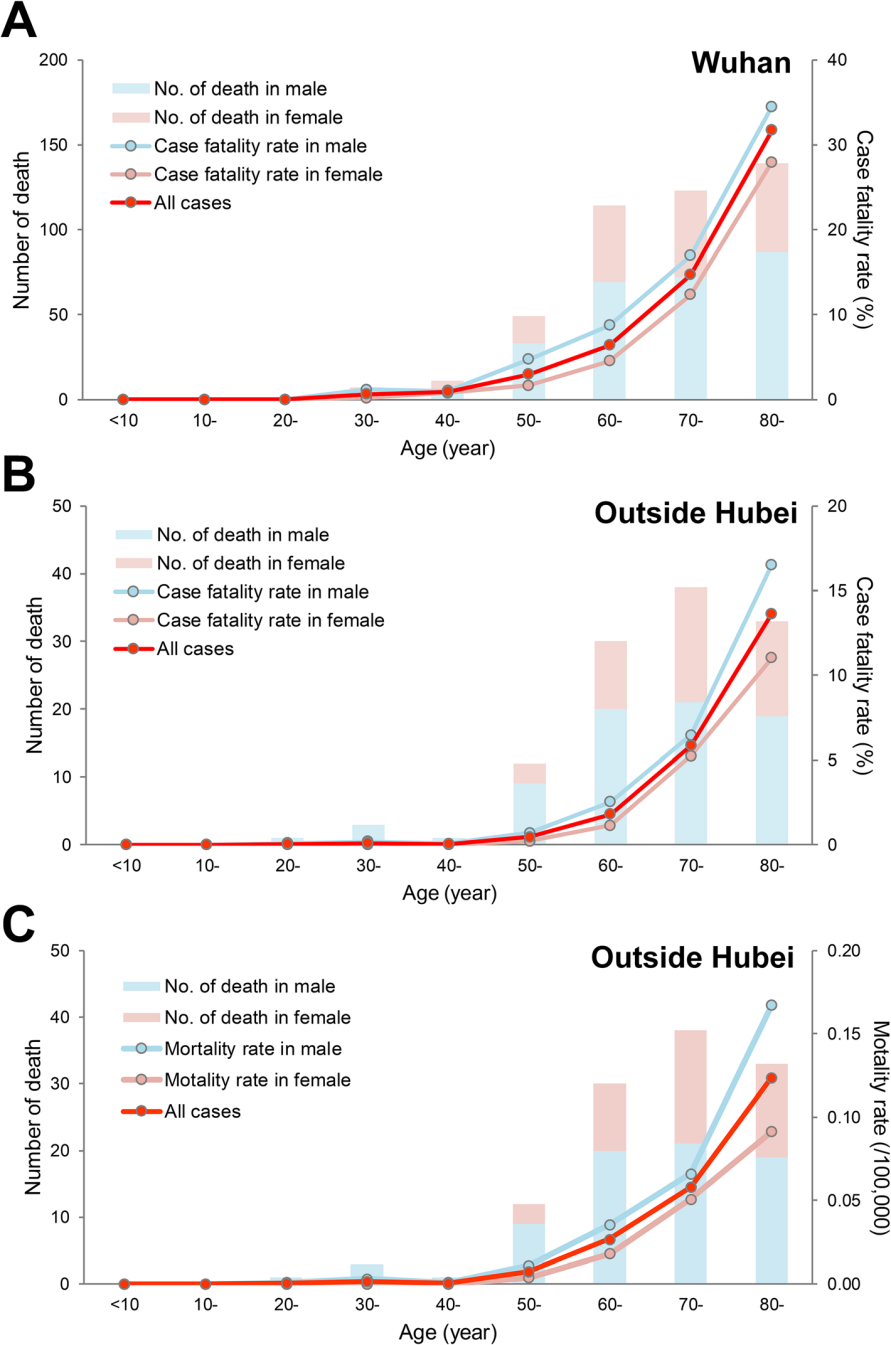
Age-specific number of deaths and hospitalized case fatality rate (HCFR) or mortality rate for COVID-19 patients by sex in different diagnosed regions in China to 29 March 2020. **a** Number of deaths and HCFR in Wuhan by a database from six hospitals in Wuhan; **b** Number of deaths and HCFR outside Hubei by a database from all the patients outside Hubei province; **c** Number of deaths and mortality rate outside Hubei, which was calculated as the number of deaths outside Hubei Province in one group formed by the intersection of age and sex divided by the number of the population outside Hubei Province in the same group obtained from population database of the National Bureau of Statistics of the People’s Republic of China in 2017

We further made a precise estimation of the age/sex HCFR that was calculated in Wuhan using the database from the six hospitals and outside Hubei Province in China. The HCFR following SARS-COV-2 infection appeared to be higher in males and increased with age; the highest HCFR observed at the male patients ≥70 years old in both regions (Fig. 1, Table S2). The outcome of COVID-19 patients was generally worse for males, but the magnitude of this difference was higher in Wuhan than in other provinces outside Hubei, with RR of death for males calculated as 1.94 (95% CI 1.59–2.36) vs. 1.49 (95% CI 1.03–2.17), respectively, compared to females (Table S3). HCFR RR also varied across age groups, which were seen in both regions. For example, in Wuhan city, the overall male to female HCFR risk ratio in all age groups was 1.94 (95% CI 1.59–2.36), with the greatest RR 2.94 (95% CI 1.60–5.38) observed for the 50–60 years of age group and the lowest RR 1.48 (95% 1.12–1.95) for the ≥70 years of age group. In a similar way, for outside Hubei Provinces, the overall male to female HCFR risk ratio in all age groups was 1.49 (95% CI 1.03–2.17), with the greatest RR 3.06 (95% CI 0.83–11.34) observed in the 50–60 years of age group, and the lowest RR 1.35 (95% CI 0.83–2.21) for the ≥70 years of age group (Table S3).

The HCFR following SARS-COV-2 infection appeared to increase with age, but with a magnitude greater in other provinces outside Hubei than in Wuhan. In Wuhan city, the age specific RR increased from 4.72 (95% CI 2.74–8.13) in the 50–60 years age group to 10.59 (95% CI 6.42–17.47) in the 60–70 years age group, to the highest RR of 40.00 (95% CI 24.68–64.84) in the ≥70 years age group. In contrast, for outside Hubei Province, a more significant effect of older age was observed: the RR increased from 7.88 (95% CI 2.77–22.40) in the 50–60 years age group, to 31.33 (95% CI 12.14–80.85) in the 60–70 years age group and to 147.37 (95% CI 59.34–366.00) in the ≥70 years age group (Table S3). The mortality rate in other provinces outside Hubei had a similar trend as that of HCFR, which was low in both sex under 40 years old, and then increased along with the increase of age, and to higher extents in female (Fig. 1c).

### Risk factor analysis for fatal outcome

**Fig. 2:**
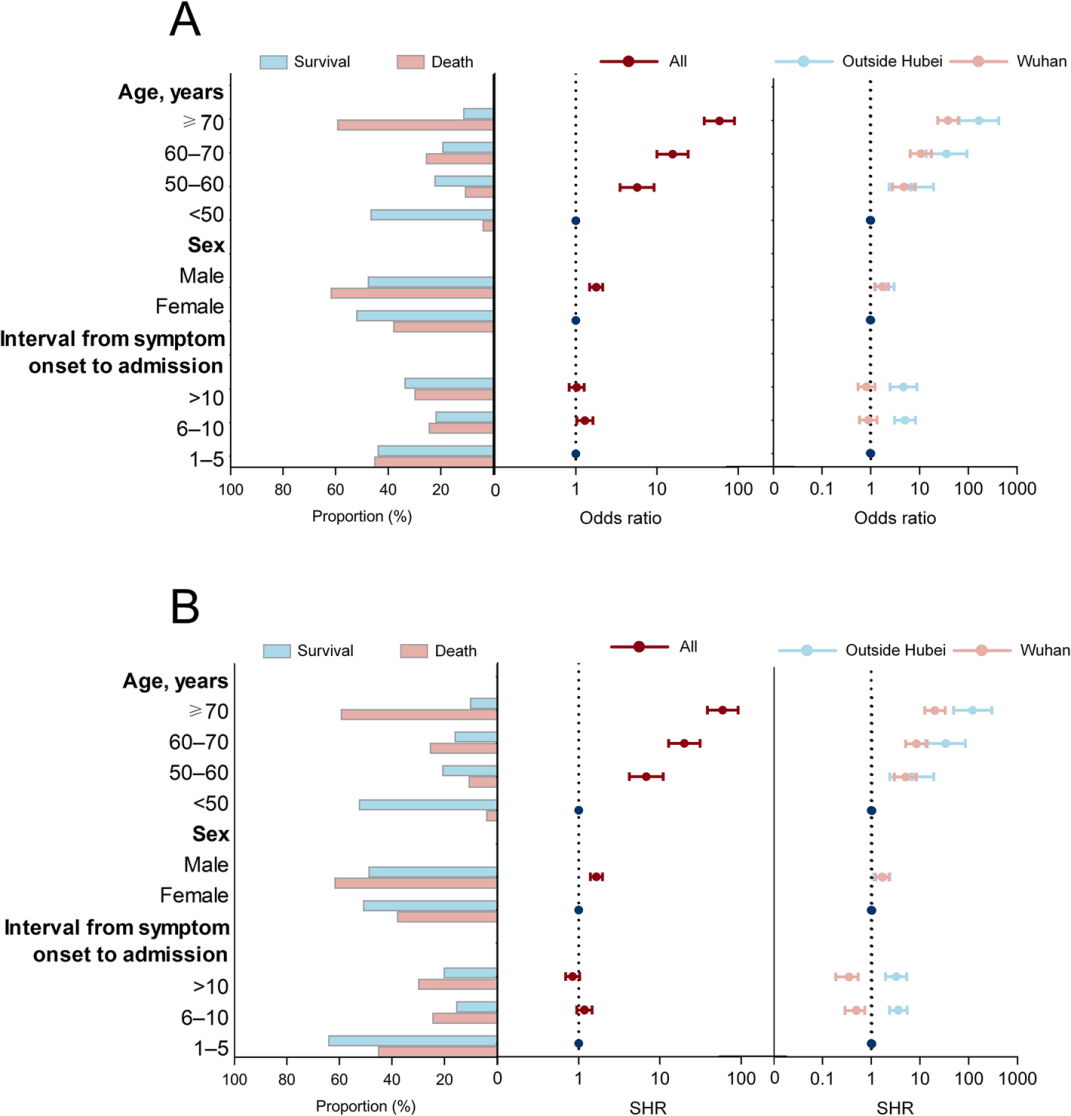
The risk factors for fatal outcome of the COVID-19 patients estimated by a logistic regression based on a case-control design (**a**) and competing risk model for fatal outcome of COVID-19 patients over time (**b**) in China to 29 March 2020. The case group included the fatal patients outside Hubei province and from six hospitals in Wuhan. The control group included the survival patients from the six hospitals and a part outside Hubei province. The proportion of patients that survived and died of COVID-19 for age and clinical intervals is shown in the left panel. In the center and right panels, the dots are odds ratios (ORs) or SHR and the error bars are 95% confidence intervals. For the model with all the patients, the OR and SHR are shown in the center column for all patients (red) and in the right panel for the patients in Wuhan (pink) and outside Hubei Province (light blue), estimated via a multivariate logistic regression model and a multivariate competing risk model. The dotted line in the center and right panels indicates an OR of 1. SHR, sub-distribution hazard ratio

In univariate and multivariate logistic regression model with a total of 10,267 survival cases and 561 fatal cases, the odds of a fatal outcome of COVID-19 patients were significantly associated with age, sex and the interval from symptom onset to admission (Table S4 and Fig. 2a). When data on fatal patients from Wuhan with 7067 survival cases and 443 fatal cases and other provinces outside Hubei Province with 3200 survival cases and 118 fatal cases were separately analyzed, the interval from symptom onset to admission were found in addition to be associated with a fatal outcome, but only in other provinces outside of Hubei and not in Wuhan. A long interval from symptom onset to admission was associated with higher HCFR, with the OR estimated to be 4.68 (95% CI 2.49–8.82) for > 10 days delay and 5.04 (95% CI 3.06–8.31) for 6–10 days compared to 1–5 days (Fig. 2a and Table S4). In all three multivariate models, age remained the strongest risk factor. Patients ≥70 years old outside Hubei had the highest risk of death, with > 167-fold increase compared to patients < 50 years old (OR = 167.05, 95% CI 65.78–424.18).

### Survival probability over time for fatal cases and related factors for clinical course

**Fig. 3:**
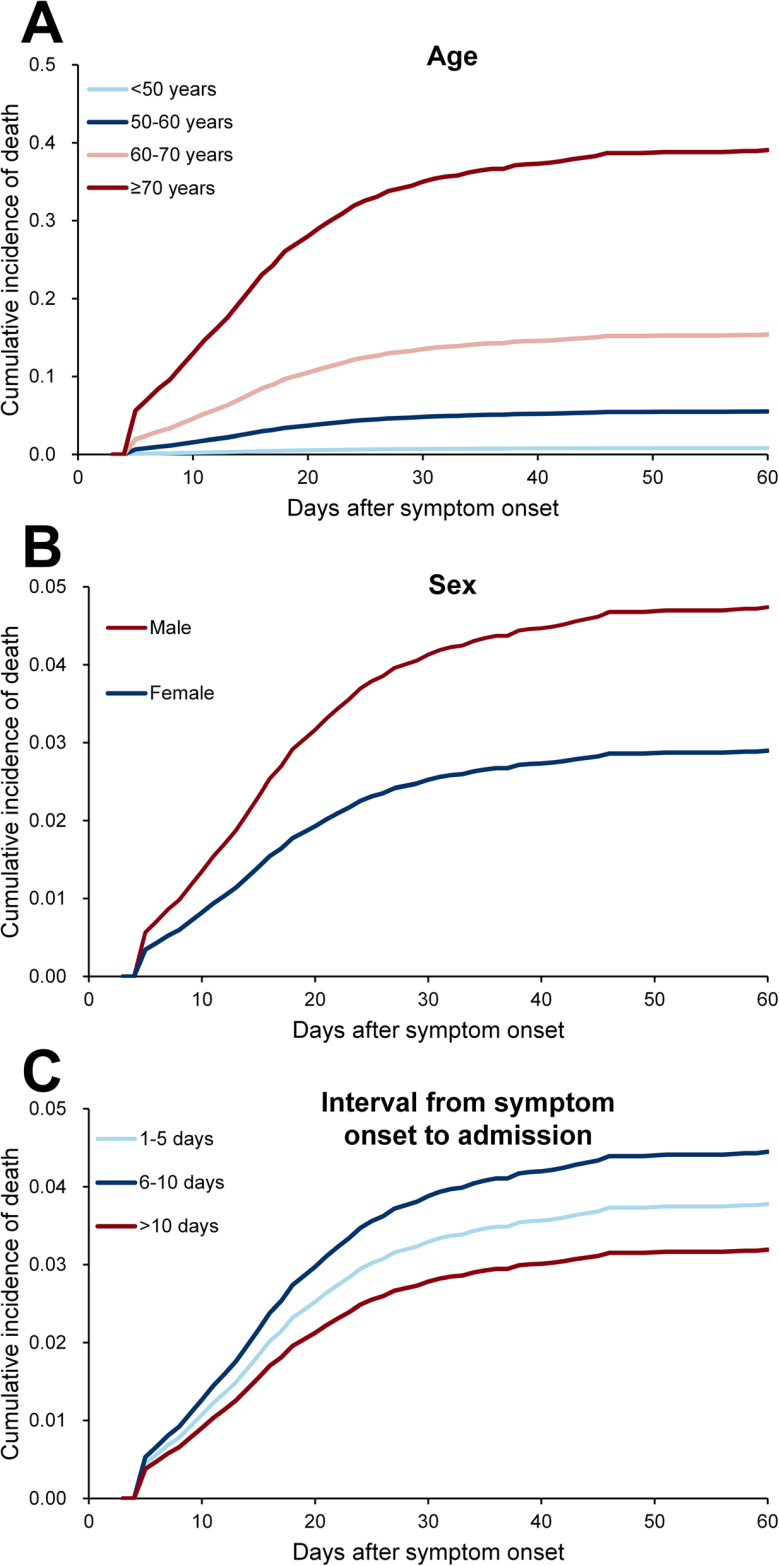
Clinical course of all COVID-19 patients with fatal and survival outcomes, and survival analysis for fatal outcome by competing risk model in China to 29 March 2020. Survival analysis based on age (< 50, 50–60, 60–70, and ≥ 70 years old) (**a**), sex (**b**) and interval from symptom onset to hospital admission (**c**). The cumulative incidence is plotted. The case group included all 118 death patients outside Hubei province and 443 deaths from six hospitals in Wuhan. The control group included the survival patients from the six hospitals (1719 cases) and a part outside Hubei province (3200 cases)

A total of 4919 survival cases (1719 in Wuhan city and 3200 outside Hubei) and 561 fatal cases (443 in Wuhan and 118 outside Hubei) were used for survival analysis. The cumulative incidence probability of survival over time for fatal cases with SARS-COV-2 infection is presented by age, sex, and the interval from symptom onset to admission (Fig. 2b and Fig. 3; Table S5). The median clinical course of the fatal cases (duration from symptom onset to death) was 15 (IQR 9–22) days. About 32.1% (130/561) and 38.3% (215/561) patients died within 1–10 days and 11–20 days after symptom onset, respectively. The median clinical course of those cases which survived (duration from symptom onset to discharge) was 23 (IQR 18–30) days. Significant differences were observed in HCFR for cases with different age and sex groups (both *P* < 0.001, Fig. 3).

By fitting a multivariate ordinal logistic regression model to fatal case data, severe patients or the patients in Wuhan city when diagnosed were associated with a shorter clinical course, indicating a very rapid progress to a fatal outcome. A longer interval from symptom onset to hospital admission was all associated with a longer clinical course (Table S6).

### Characteristics of fatal patients with mild pneumonia

There was 39.6% (222/561) of fatal cases who were admitted to hospital with mild pneumonia; the remainder 60.4% were admitted with severe pneumonia (Table S7). The median age of fatal cases admitted as mild was 75 (IQR 65–82) years, comparable with 72 (IQR 64–80) years for severe cases. The sex distribution was also comparable. Both the interval from symptom onset to diagnosis and the interval from symptom onset to admission were comparable between fatal cases with mild pneumonia and those with severe pneumonia.

### The temporal pattern of HCFR

**Fig. 4:**
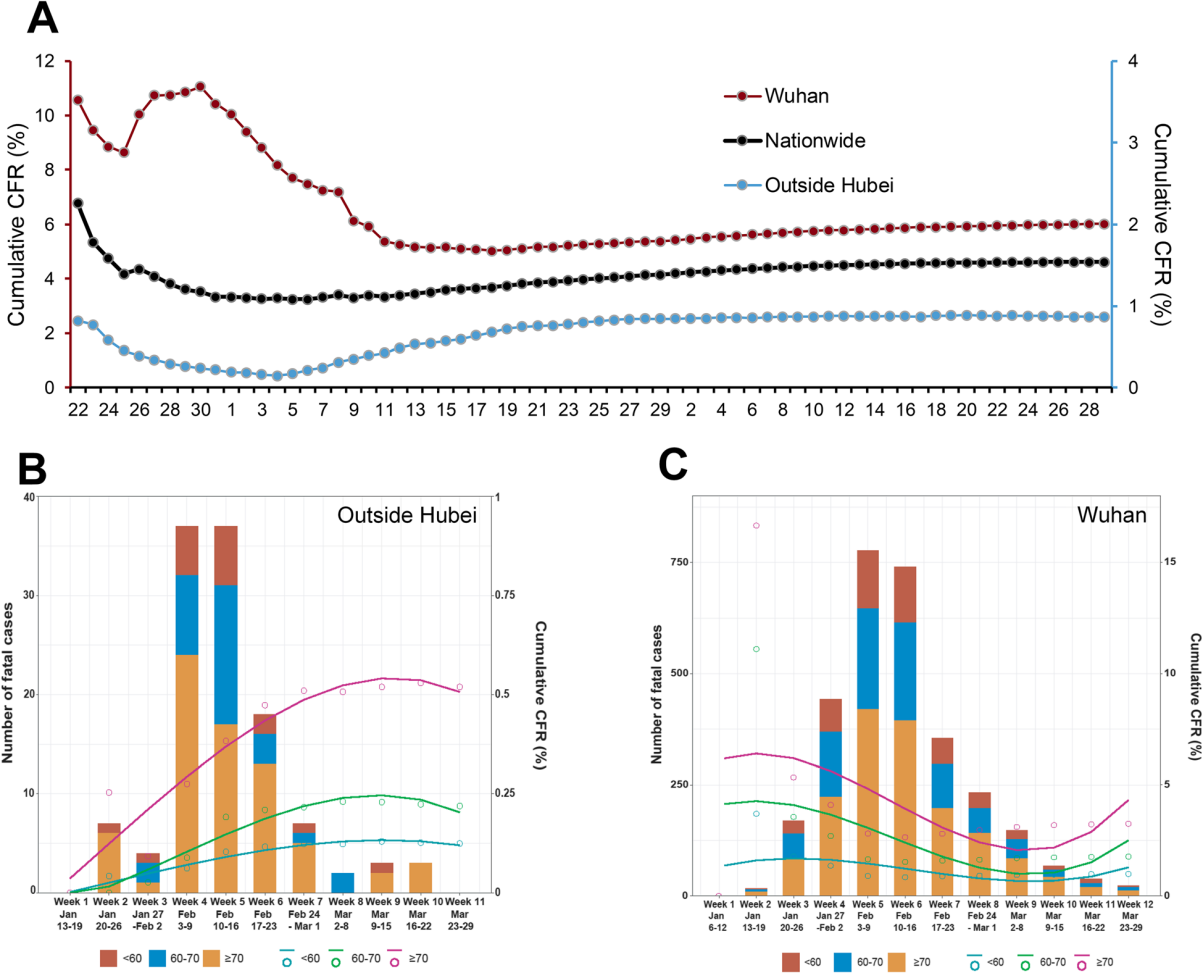
Cumulative HCFRs of COVID-19 patients by reported dates in nationwide, Wuhan and outside Hubei Province in China to 29 March 2020. **a** Cumulative HCFRs of COVID-19 patients in nationwide, Wuhan and outside Hubei Province by reported weeks; The left Y axis was used for nationwide and Wuhan, and the right Y axis was used for outside Hubei. **b** Cumulative HCFRs outside Hubei in different age groups by a database of all the patients outside Hubei. **c** Cumulative HCFRs in Wuhan in different age groups by a database of all the patients in Wuhan. HCFR, hospitalized case fatality rate. The case group included the fatal patients outside Hubei province and from six hospitals in Wuhan. The control group included the survival patients from the six hospitals and a part outside Hubei province

The daily cumulative HCFR was estimated by using the reported number of total fatal cases divided by the daily number of total confirmed cases reported (Fig. 4). For Wuhan fatal cases, the HCFR in January was significantly higher than during the later phase of the epidemic (Fig. 4a). There was a clear peak in HCFR during the week of 23–29 January 2020, followed by an obvious reduction thereafter to a stable level until recently. The national temporal trend was dominated by that of Wuhan city where both the total number of cases and fatal cases outweighs the other provinces (Fig. 4a). In contrast, for provinces outside Hubei, there was an obvious low HCFR observed during the first week of February, a period which corresponded to 1 week after Chinese spring festival travel rush; a tender increase was followed, bringing the HCFR to the peaking level at the end of February (Week 6 of the epidemic); a decreasing trend was then observed after February, where the HCFR was maintained at a stable level till the latest observation (Fig. 4a).

The longitudinal HCFR profiles were compared among different age groups and between Wuhan and outside Hubei Province. Generally, the HCFR curve of three groups showed similar dynamic trends, but with much greater magnitude in the ≥70 years group than the other two age groups. For example, both the decrease of HCFR at the end of February in Wuhan and the increase of HCFR at the early February in other provinces outside Hubei were more obvious and notable in the ≥70 years group than the other two age groups, remarkably contributing to the overall trend of HCFR in both regions (Fig. 4b, c).

## Discussion

Assessing the mortality rate of emerging infections is challenging because of potential biases in case ascertainment and delays in counting the occurrence of deaths. This dilemma is rather obvious for COVID-19, where the estimated CFR differs between countries/regions, age, sex, and numerous other patient factors [20–25]. In China, published CFR data is often presented as Wuhan data versus non-Wuhan data. As the initial epicenter of COVID-19, Wuhan experienced the chaos of changing diagnostic criteria and standard treatment regimens. Therefore, it is not surprising that large CFR discrepancies have been reported in Wuhan, especially during the early epidemic phase. The national official statistics reported a CFR of 2.3% in China out of 44,673 cases as of 11 February 2020 [7]; however, as with some other reports, each patient’s eventual outcome was not determined.

Here, based on the most recently released data as end of March 2020, the overall HCFR in China is 4.6%, HCFR increased with age and was higher in males than in females, the highest HCFR being observed in male patients ≥70 years of age. We also identified major disparities in age- and sex-specific rates of HCFR from SARS-CoV-2 in Wuhan versus other regions, the effect of age on HCFR being greater outside Hubei provinces than Wuhan. The effect of longer interval from symptom onset and hospital admission was only observed outside Hubei Province. We suggest that the effect of these two variables were partially masked by the effect of differential medical care received between regions. Although no specific anti-viral therapy is available yet, supportive care is crucial for survival from severe respiratory disease.

Until recently, the male-female differences in COVID-19 morbidity and mortality have remained rarely investigated, especially regarding the differences within different age groups. Here we inferred detailed age- and sex-associated differences in HCFR during the epidemic, which might reflect many different biological and behavioral factors. Although the outcome of infection is generally worse for males, this risk decreased in older age groups. Overall a poorer outcome of infection was seen as age increases, but this effect was more pronounced for females than males. Indeed, older age was consistently a risk for higher HCFR, regardless of any other influencing factors, such as sex or co-morbidities [26, 27]. Still the highest risk of death was observed for male patients ≥70 years of age, regardless of their geographic location.

Although the incidence data showed an approximately equal number of male and female cases, more men than women suffered from severe disease and died [21, 28]. The data from other countries demonstrated similar results [29]. Adverse outcomes of COVID-19 have been associated with comorbidities, including hypertension, cardiovascular disease, and lung disease. These conditions are more prevalent in men and linked to behaviors such as smoking and drinking alcohol [29]. Sex-based immunological differences have also been suggested as another potential explanation [3]. During the pandemics, women were more likely to practice non-pharmaceutical behaviors - such as hand washing, face mask use and avoiding crowds - compared to men [30], which may be in part responsible for the differences observed.

The current study also found a differential outbreak pattern between Wuhan and provinces outside Hubei. For Wuhan city, early in the outbreak, it is possible that more of the severe cases were detected and tested. High clinical volumes and underestimation of confirmed cases early in the epidemic might have contributed to this higher mortality rate in Wuhan. This surge might not reflect rapid death from severe disease, but rather might be due to the backlog in diagnostic confirmation caused by limited supplies and laboratory facilities early during the epidemic. Since 25 January, tests have become increasingly available for clinically suspected patients. The HCFR calculated from Wuhan continued to decrease after a brief surge, however it remained higher than other provinces throughout the epidemic. Age, although reported to be an important factor in determining the risk of a fatal outcome, was not responsible for the higher HCFR in Wuhan because the age effect was controlled in multivariate analysis. Rather, the higher HCFR in Wuhan might be explained by case ascertainment. With thousands of serious cases, mild or asymptomatic cases that might account for most SARS-COV-2 infections would likely remain largely unrecognized. Accordingly, the official numbers of both cases and deaths reported from Wuhan represent the “tip of the iceberg”, potentially skewing HCFR estimates towards patients presenting with more severe disease and a fatal outcome. We can infer that HCFR truly differed between regions. The differences in medical care during a large epidemic versus care for single cases could be responsible for the differences in case fatality rates observed.

Whereas in other provinces early fatal cases were mainly attributed to imported sources with younger patient age and lower HCFR, later on the fatal cases were mainly attributed to local transmission with higher HCFR. We propose that there might be a higher HCFR in local transmission patients than those in imported case clusters, which has also been suggested in previous studies [31]. Except for these two minor fluctuations, no reduction in HCFR was observed for either of the regions until the end of the epidemic.

A small proportion of fatal patients exhibited only mild pneumonia, and this was more common in Wuhan. The mild disease in Wuhan was also associated with a rapid clinical progression to death, compared to those presenting with severe pneumonia. Consistent with the findings of Guan et al. 23.87% of severe cases had no abnormal radiological findings, indicating that a severe outcome can occur even without associated severe lung pathology [10]. In the study by Chen et al., SARS-COV-2 infection triggered a cytokine storm during the acute phase of disease that was related to an adverse outcome [3]. This inflammatory reaction can cause very severe disease without extensive lung damage; also, such case progression might not be detected, during hospitalization.

This study was subject to the limitation that only hospitalized patients were included in the estimation. As described in previous studies, SARS-COV-2 infection can manifest as asymptomatic self-limited disease. In the absence of asymptomatically infected cases, or very mild cases of infection detected via immunology testing, we can only obtain an estimation of the hospitalized CFR. We acknowledge that the fatal outcome of COVID-19 should be associated with the underlying medical conditions of the enrolled patients, including cardio-vascular diseases, diabetes, cancer, et al. however, the current dataset has not included these factors for analysis, as the study was not designed at hospital level. In this study, the effects of age and sex on the fatal outcome of COVID-19 were mainly examined, and the unavailability of detailed medical records has held the analysis on more COVID-19 risk factors back. Future studies are needed by using medical records regarding the whole course of disease for COVID-19 patients at hospital level.

## Conclusions

The current study offers a detailed view on case fatality rate in relation to epidemiological data from both an epidemic center in which SARS-COV-2 transmission was intense and from regions in which the epidemic was well controlled. Epidemic curves from Wuhan and from provinces outside Hubei demonstrate the common feature of age as the main risk factor for HCFR, especially among male patients, and that HCFR remained relatively stable at the late epidemic. Differential demographic characteristics were found for the Wuhan fatal cases: 1) less effect on HCFR at older ages or longer hospital admission delay, and 2) higher frequency of mild disease on admission to hospital. These findings might be explained by the profound differences in diagnostic capacity, health seeking behaviors and health care in Wuhan. This up-to-date and comprehensive description of COVID-19 HCFR and its drivers will help healthcare providers to target limited medical resources to patients with high risks of a fatal outcome.

## Supplementary Information

**Additional file 1: Figure S1**. Geographical distribution of case fatality rate of the COVID-19 patients based on the surveillance database in the mainland of China as of 29 March 2020. **Table S1**. The hospitalized case fatality rates of COVID-19 patients in related provinces. **Table S2**. The mortality rate and hospitalized case fatality rate of COVID-19 in China. **Table S3**. The hospitalized case fatality rate and relative risk for death outcome of COVID-19 patients by location, age, and sex. **Table S4**. The risk factor of fatal outcome among confirmed patients with SARS-COV-2 infection in the case-control study. **Table S5**. The cumulative incidence probability of death over time for the COVID-19 patients. **Table S6**. Clinical course of fatal cases and the factors associated with death among confirmed patients with SARS-COV-2 infection. **Table S7**. Comparison on the characteristics of fatal cases with SARS-COV-2 infection between severe and mild cases as of 29 March 2020.

## Data Availability

The datasets used and/or analysed during the current study are available from the corresponding author on reasonable request.

## Abbreviations

COVID-19: Coronavirus disease 2019
SARS-CoV-2: Severe acute respiratory syndrome coronavirus 2
CFR: Case fatality rate
HCFR: Hospitalized CFR
SHR: Sub-distribution hazard ratio
95% CI: 95% confidence interval
