# Supplementary material for: The differential demographic pattern of coronavirus disease 2019 fatality outside Hubei and from six hospitals in Hubei, China: a descriptive analysis
Source: BMC Infect Dis. 2021 May 26;21:481. doi: 10.1186/s12879-021-06187-4 (PMC8153527; doi:10.1186/s12879-021-06187-4)
Supplement: Supplementary file 1 — Additional file 1: Figure S1. Geographical distribution of case fatality rate of the COVID-19 patients based on the surveillance database in the mainland of China as of 29 March 2020. Table S1. The hospitalized case fatality rates of COVID-19 patients in related provinces. Table S2. The mortality rate and hospitalized case fatality rate of COVID-19 in China. Table S3. The hospitalized case fatality rate and relative risk for death outcome of COVID-19 patients by location, age, and sex. Table S4. The risk factor of fatal outcome among confirmed patients with SARS-COV-2 infection in the case-control study. Table S5. The cumulative incidence probability of death over time for the COVID-19 patients. Table S6. Clinical course of fatal cases and the factors associated with death among confirmed patients with SARS-COV-2 infection. Table S7. Comparison on the characteristics of fatal cases with SARS-COV-2 infection between severe and mild cases as of 29 March 2020. [file 12879_2021_6187_MOESM1_ESM.docx]

**Appendix To**

The differential demographic pattern of fatal patients with corona virus disease 2019 in Wuhan and Other Provinces in China: A descriptive analysis

**Figure S1. Geographical distribution of case fatality rate of the COVID-19 patients based on the surveillance database in the mainland of China as of 29 March 2020.**

A, the hospitalized case fatality rate (HCFR) in China; B, the HCFR in Hubei Province; C, the case fatality rate in Wuhan city; D, the mortality rate in Hubei Province; E, the mortality rate in Wuhan city.


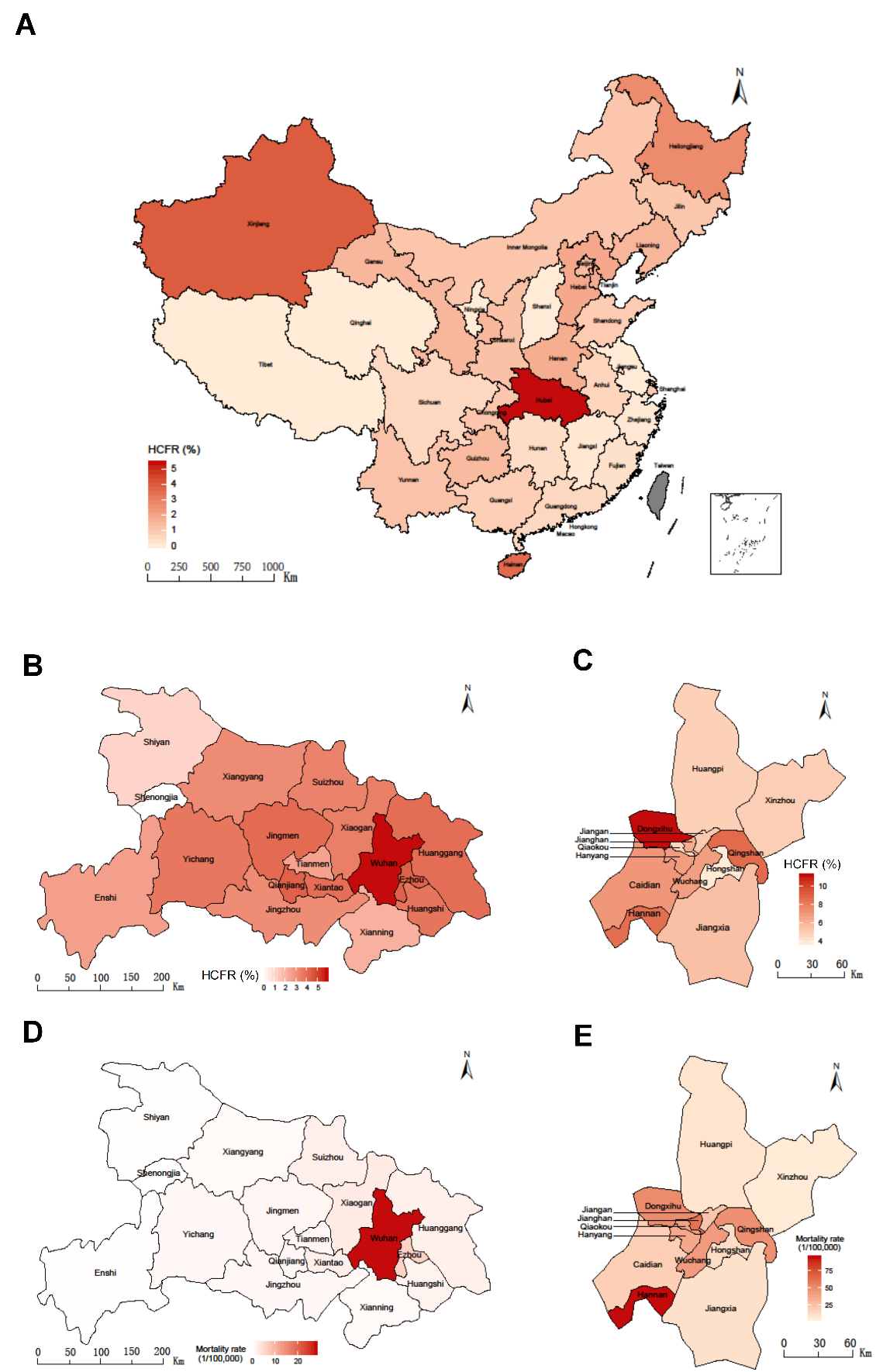


**Table S1. The hospitalized case fatality rates of COVID-19 patients in related provinces**

| **Province** | **No. of fatal cases** | **All confirmed cases** | **HCFR (%)** | **95% CI** |
| --- | --- | --- | --- | --- |
| Total outside Hubei | 118 | 13669 | 0.86 | 0.72‒1.03 |
| Xinjiang | 3 | 76 | 3.95 | 0.82‒11.11 |
| Hainan | 6 | 168 | 3.57 | 1.32‒7.61 |
| Heilongjiang | 13 | 486 | 2.67 | 1.43‒4.53 |
| Hebei | 6 | 321 | 1.87 | 0.69‒4.02 |
| Tianjin | 3 | 167 | 1.80 | 0.37‒5.16 |
| Henan | 22 | 1,276 | 1.72 | 1.08‒2.60 |
| Liaoning | 2 | 136 | 1.47 | 0.18‒5.21 |
| Gansu | 2 | 138 | 1.45 | 0.18‒5.14 |
| Beijing | 8 | 579 | 1.38 | 0.60‒2.70 |
| Guizhou | 2 | 147 | 1.36 | 0.17‒4.83 |
| Shaanxi | 3 | 253 | 1.19 | 0.25‒3.43 |
| Yunnan | 2 | 180 | 1.11 | 0.13‒3.96 |
| Inner Mongolia | 1 | 95 | 1.05 | 0.03‒5.73 |
| Chongqing | 6 | 579 | 1.04 | 0.38‒2.24 |
| Jilin | 1 | 98 | 1.02 | 0.03‒5.55 |
| Shanghai | 5 | 499 | 1.00 | 0.33‒2.32 |
| Shandong | 7 | 773 | 0.91 | 0.36‒1.86 |
| Guangxi | 2 | 254 | 0.79 | 0.10‒2.82 |
| Anhui | 6 | 990 | 0.61 | 0.22‒1.31 |
| Sichuan | 3 | 550 | 0.55 | 0.11‒1.59 |
| Guangdong | 8 | 1,484 | 0.54 | 0.23‒1.06 |
| Hunan | 4 | 1,018 | 0.39 | 0.11‒1.00 |
| Fujian | 1 | 340 | 0.29 | 0.01‒1.63 |
| Jiangxi | 1 | 932 | 0.11 | 0‒0.60 |
| Zhejiang | 1 | 1,255 | 0.08 | 0‒0.44 |
| Jiangsu | 0 | 136 | 0 | 0‒2.68 |
| Shanxi | 0 | 645 | 0 | 0‒0.57 |
| Ningxia | 0 | 1 | 0 | 0‒97.5 |
| Qinghai | 0 | 18 | 0 | 0‒18.53 |
| Tibet | 0 | 75 | 0 | 0‒4.80 |
| Hubei | 3,651 | 67,801 | 5.38 | 5.22‒5.56 |

HCFR, hospitalized case fatality rates; CI, confidence interval; COVID-19, coronavirus disease 2019.

**Table S2. The mortality rate and hospitalized case fatality rate of COVID-19 in China**

| **Age** | **HCFR in Wuhan (%)** | | | |  | **HCFR outside Hubei (%)** | | | |  | **Mortality rate (/100,000)** | | | |
| --- | --- | --- | --- | --- | --- | --- | --- | --- | --- | --- | --- | --- | --- | --- |
|  | **Total** | **Male** | **Female** | **P** |  | **Total** | **Male** | **Female** | **P** |  | **Total** | **Male** | **Female** | **P** |
| <10 | 0 | 0 | 0 | 1.000 |  | 0 | 0 | 0 | 1.000 |  | 0 | 0 | 0 | 1.000 |
| 10‒ | 0 | 0 | 0 | 1.000 |  | 0 | 0 | 0 | 1.000 |  | 0 | 0 | 0 | 1.000 |
| 20‒ | 0 | 0 | 0 | 1.000 |  | 0.05 | 0.10 | 0 | 1.000 |  | 0.0004 | 0.0009 | 0 | 1.000 |
| 30‒ | 0.64 | 1.18 | 0.17 | 0.056 |  | 0.10 | 0.19 | 0 | 0.257 |  | 0.0016 | 0.0031 | 0 | 0.250 |
| 40‒ | 0.91 | 1.04 | 0.79 | 0.647 |  | 0.03 | 0.06 | 0 | 1.000 |  | 0.0004 | 0.0008 | 0 | 1.000 |
| 50‒ | 2.96 | 4.75 | 1.67 | <0.001 |  | 0.46 | 0.70 | 0.23 | 0.090 |  | 0.0075 | 0.0110 | 0.0038 | 0.147 |
| 60‒ | 6.41 | 8.73 | 4.55 | 0.001 |  | 1.81 | 2.54 | 1.15 | 0.037 |  | 0.0269 | 0.0356 | 0.018 | 0.074 |
| 70‒ | 14.70 | 16.94 | 12.38 | 0.122 |  | 5.86 | 6.46 | 5.25 | 0.534 |  | 0.0583 | 0.0661 | 0.0509 | 0.420 |
| 80‒ | 31.74 | 34.52 | 27.96 | 0.291 |  | 13.64 | 16.52 | 11.02 | 0.278 |  | 0.1237 | 0.1673 | 0.0914 | 0.081 |
| Overall | 5.90 | 7.86 | 4.21 | <0.001 |  | 0.86 | 1.02 | 0.69 | 0.036 |  | 0.0089 | 0.0109 | 0.0068 | 0.012 |

HCFR, hospitalized case fatality rates; COVID-19, coronavirus disease 2019.

**Table S3. The hospitalized case fatality rate and relative risk for death outcome of COVID-19 patients by location, age, and sex**

| **Group** | **HCFR**  **n (%)** | **RR**  **(95% CI)** | **P value** |
| --- | --- | --- | --- |
| **Wuhan** | 443 (5.90) | - | - |
| Age group |  |  |  |
| <50 | 18 (0.64) | Reference |  |
| 50‒60 | 49 (2.96) | 4.72 (2.74‒8.13) | <0.001 |
| 60‒70 | 114 (6.41) | 10.59 (6.42‒17.47) | <0.001 |
| ≥70 | 262 (20.55) | 40.00 (24.68‒64.84) | <0.001 |
| <50 |  |  |  |
| Male | 12 (0.91) | 2.28 (0.85‒6.09) | 0.100 |
| Female | 6 (0.40) | Reference |  |
| 50‒60 |  |  |  |
| Male | 33 (4.75) | 2.94 (1.60‒5.38) | <0.001 |
| Female | 16 (1.67) | Reference |  |
| 60‒70 |  |  |  |
| Male | 69 (8.73) | 2.01 (1.36‒2.96) | <0.001 |
| Female | 45 (4.55) | Reference |  |
| ≥70 |  |  |  |
| Male | 159 (23.49) | 1.48 (1.12‒1.95) | 0.006 |
| Female | 103 (17.22) | Reference |  |
| Sex |  |  |  |
| Male | 273 (7.86) | 1.94 (1.59‒2.36) | <0.001 |
| Female | 170 (4.21) | Reference |  |
| Male |  |  | <0.001 |
| <50 | 12 (0.91) | Reference |  |
| 50‒60 | 33 (4.75) | 5.40 (2.77‒10.53) | <0.001 |
| 60‒70 | 69 (8.73) | 10.38 (5.58‒19.28) | <0.001 |
| ≥70 | 159 (23.49) | 33.28 (18.35‒60.37) | <0.001 |
| Female |  |  |  |
| <50 | 6 (0.40) | Reference |  |
| 50‒60 | 16 (1.67) | 4.19 (1.64‒10.76) | 0.003 |
| 60‒70 | 45 (4.55) | 11.78 (5.01‒27.72) | <0.001 |
| ≥70 | 103 (17.22) | 51.43 (22.44‒117.88) | <0.001 |
| **Outside Hubei** | 118 (0.86) | **-** | **-** |
| Age group |  |  |  |
| <50 | 5 (0.06) | Reference |  |
| 50‒60 | 12 (0.46) | 7.88 (2.77‒22.40) | <0.001 |
| 60‒70 | 30 (1.81) | 31.33 (12.14‒80.85) | <0.001 |
| ≥70 | 71 (7.97) | 147.37 (59.34‒366) | <0.001 |
| <50 |  |  |  |
| Male | 5 (0.11) | - | 0.070 |
| Female | 0 (0) | - |  |
| 50‒60 |  |  |  |
| Male | 9 (0.70) | 3.06 (0.83‒11.34) | 0.094 |
| Female | 3 (0.23) | Reference |  |
| 60‒70 |  |  |  |
| Male | 20 (2.54) | 2.24 (1.05‒4.83) | 0.038 |
| Female | 10 (1.15) | Reference |  |
| ≥70 |  |  |  |
| Male | 40 (9.09) | 1.35 (0.83‒2.21) | 0.223 |
| Female | 31 (6.87) | Reference |  |
| Sex |  |  |  |
| Male | 74 (1.02) | 1.49 (1.03‒2.17) | 0.036 |
| Female | 44 (0.69) | Reference |  |
| Male |  |  |  |
| <50 | 5 (0.11) | Reference |  |
| 50‒60 | 9 (0.70) | 6.64 (2.22‒19.85) | 0.001 |
| 60‒70 | 20 (2.54) | 24.63 (9.22‒65.82) | <0.001 |
| ≥70 | 40 (9.09) | 94.58 (37.12‒240.98) | <0.001 |
| Female |  |  |  |
| <50 | 0 (0) | - |  |
| 50‒60 | 3 (0.23) | Reference |  |
| 60‒70 | 10 (1.15) | 5.06 (1.39‒18.45) | 0.014 |
| ≥70 | 31 (6.87) | 32.21 (9.80‒105.88) | <0.001 |

HCFR, hospitalized case fatality rates; COVID-19, coronavirus disease 2019; RR, relative risk; CI, confidence interval

**Table S4. The risk factor of fatal outcome among confirmed patients with SARS-COV-2 infection in the case-control study**

| **Characteristic** | **Survival** | **Fatal** | **Univariate** | |  | **Multivariate** | |
| --- | --- | --- | --- | --- | --- | --- | --- |
|  |  |  | **OR (95%CI)** | **P** |  | **OR (95%CI)** | **P** |
| **Total, n (%)** | 10267 (94.82) | 561 (5.18) |  |  |  |  |  |
| Age, years, n (%) |  |  |  |  |  |  |  |
| <50 | 4800 (46.8) | 23 (4.1) | Reference |  |  | Reference |  |
| 50‒ | 2300 (22.4) | 61 (10.9) | 5.53 (3.42‒8.96) | <0.001 |  | 5.7 (3.52‒9.24) | <0.001 |
| 60‒ | 1990 (19.4) | 144 (25.7) | 15.1 (9.69‒23.52) | <0.001 |  | 15.54 (9.96‒24.25) | <0.001 |
| 70‒ | 1177 (11.5) | 333 (59.4) | 59.04 (38.51‒90.53) | <0.001 |  | 58.7 (38.25‒90.09) | <0.001 |
| Sex, n (%) |  |  |  |  |  |  |  |
| Female | 5355 (52.2) | 214 (38.1) | Reference |  |  | Reference |  |
| Male | 4912 (47.8) | 347 (61.9) | 1.77 (1.48‒2.11) | <**0.**001 |  | 1.78 (1.48‒2.14) | <0.001 |
| Interval from symptom onset to admission, days, n (%) |  |  |  |  |  |  |  |
| 1‒5 | 5546 (54) | 255 (45.5) | Reference |  |  | Reference |  |
| 6‒10 | 1929 (18.8) | 137 (24.4) | 1.54 (1.25‒1.91) | <0.001 |  | 1.29 (1.03‒1.63) | 0.027 |
| >10 | 2792 (27.2) | 169 (30.1) | 1.32 (1.08‒1.61) | 0.007 |  | 1.03 (0.83‒1.27) | 0.781 |
| **Wuhan, n (%)** | 7067 (94.10) | 443 (5.90) |  |  |  |  |  |
| Age, years, n (%) |  |  |  |  |  |  |  |
| <50 | 2784 (39.4) | 18 (4.1) | Reference |  |  | Reference |  |
| 50‒ | 1605 (22.7) | 49 (11.1) | 4.72 (2.74‒8.13) | <0.001 |  | 4.92 (2.85‒8.48) | <0.001 |
| 60‒ | 1665 (23.6) | 114 (25.7) | 10.59 (6.42‒17.47) | <0.001 |  | 10.9 (6.6‒18) | <0.001 |
| 70‒ | 1013 (14.3) | 262 (59.1) | 40 (24.68‒64.84) | <0.001 |  | 39.25 (24.21‒63.65) | <0.001 |
| Sex, n (%) |  |  |  |  |  |  |  |
| Female | 3865 (54.7) | 170 (38.4) | Reference |  |  | Reference |  |
| Male | 3202 (45.3) | 273 (61.6) | 1.94 (1.59‒2.36) | <0.001 |  | 1.78 (1.44‒2.18) | <0.001 |
| Interval from symptom onset to admission, days, n (%) |  |  |  |  |  |  |  |
| 1‒5 | 2790 (39.5) | 196 (44.2) | Reference |  |  | Reference |  |
| 6‒10 | 1612 (22.8) | 99 (22.4) | 0.87 (0.68‒1.12) | 0.291 |  | 0.91 (0.7‒1.19) | 0.503 |
| >10 | 2660 (37.7) | 148 (33.4) | 0.79 (0.64‒0.99) | 0.038 |  | 0.83 (0.66‒1.05) | 0.119 |
| **Outside Hubei, n (%)** | 3200 (87.25) | 118 (12.75) |  |  |  |  |  |
| Age, years, n (%) |  |  |  |  |  |  |  |
| <50 | 2016 (63) | 5 (4.2) | Reference |  |  | Reference |  |
| 50‒ | 695 (21.7) | 12 (10.2) | 6.96 (2.44‒19.83) | <0.001 |  | 6.78 (2.37‒19.41) | <0.001 |
| 60‒ | 325 (10.2) | 30 (25.4) | 37.22 (14.34‒96.62) | <0.001 |  | 35.81 (13.68‒93.72) | <0.001 |
| 70‒ | 164 (5.1) | 71 (60.2) | 174.56 (69.51‒438.33) | <0.001 |  | 167.05 (65.78‒424.18) | <0.001 |
| Sex, n (%) |  |  |  |  |  |  |  |
| Female | 1490 (46.6) | 44 (37.3) | Reference |  |  | Reference |  |
| Male | 1710 (53.4) | 74 (62.7) | 1.47 (1‒2.14) | 0.048 |  | 1.93 (1.25‒2.99) | 0.003 |
| Interval from symptom onset to admission, days, n (%) |  |  |  |  |  |  |  |
| 1‒5 | 2751 (86) | 59 (50) | Reference |  |  | Reference |  |
| 6‒10 | 317 (9.9) | 38 (32.2) | 5.59 (3.66‒8.54) | <0.001 |  | 5.04 (3.06‒8.31) | <0.001 |
| >10 | 132 (4.1) | 21 (17.8) | 7.42 (4.38‒12.57) | <0.001 |  | 4.68 (2.49‒8.82) | <0.001 |

**Table S5. The cumulative incidence probability of death over time for the COVID-19 patients**

| Characteristics | Survival | |  | Death | | SHR (95% CI)* | P |
| --- | --- | --- | --- | --- | --- | --- | --- |
|  | No. of cases (%) | Median (IQR) |  | No. of cases (%) | Median (IQR) |  |  |
| **Total** | 4919 (89.8) | 23 (18‒30) |  | 561 (10.2) | 15 (9‒22) |  |  |
| Age, years |  |  |  |  |  |  |  |
| <50 | 2586 (52.6) | 21 (16‒27) |  | 23 (4.1) | 14 (11‒17) | Reference |  |
| 50‒60 | 1029 (20.9) | 23 (18‒30) |  | 61 (10.9) | 16 (10‒24) | 6.79 (4.2‒10.97) | <0.001 |
| 60‒70 | 799 (16.2) | 27 (21‒35) |  | 144 (25.7) | 15 (9‒24) | 20.05 (12.84‒31.32) | <0.001 |
| ≥70 | 505 (10.3) | 28 (22‒37) |  | 333 (59.4) | 14 (9‒21) | 59.45 (38.54‒91.7) | <0.001 |
| Sex |  |  |  |  |  |  |  |
| Male | 2510 (51) | 23 (17‒29) |  | 347 (61.9) | 15 (9‒23) | 1.65 (1.39‒1.96) | <0.001 |
| Female | 2409 (49) | 23 (18‒30) |  | 214 (38.1) | 15 (8‒22) | Reference |  |
| Interval from symptom onset to admission, days |  |  |  |  |  |  |  |
| 1‒5 | 3161 (64.3) | 20 (16‒25) |  | 255 (45.5) | 9 (5‒18) | Reference |  |
| 6‒10 | 762 (15.5) | 26 (21‒30) |  | 137 (24.4) | 15 (11‒22) | 1.18 (0.95‒1.46) | 0.126 |
| >10 | 996 (20.3) | 34 (27‒40) |  | 169 (30.1) | 20 (15‒26) | 0.84 (0.69‒1.03) | 0.090 |
| **Wuhan** | 1719 (79.5) | 29 (23‒37) |  | 443 (20.5) | 14 (9‒22) |  |  |
| Age, years |  |  |  |  |  |  |  |
| <50 | 570 (33.2) | 27 (22‒34) |  | 18 (4.1) | 14 (10‒16) | Reference |  |
| 50‒60 | 334 (19.4) | 29 (23‒37) |  | 49 (11.1) | 16 (10‒24) | 4.94 (2.86‒8.5) | <0.001 |
| 60‒70 | 474 (27.6) | 32 (26‒38) |  | 114 (25.7) | 15 (8‒23) | 8.17 (4.95‒13.5) | <0.001 |
| ≥70 | 341 (19.8) | 32 (25‒40) |  | 262 (59.1) | 14 (9‒21) | 19.59 (12.08‒31.76) | <0.001 |
| Sex |  |  |  |  |  |  |  |
| Male | 800 (46.5) | 29 (23‒37) |  | 273 (61.6) | 14 (9‒21) | 1.68 (1.38‒2.04) | <0.001 |
| Female | 919 (53.5) | 30 (24‒37) |  | 170 (38.4) | 14 (8‒22) | Reference |  |
| Interval from symptom onset to admission, days |  |  |  |  |  |  |  |
| 1‒5 | 410 (23.9) | 22 (17‒27) |  | 196 (44.2) | 9 (5‒17) | Reference |  |
| 6‒10 | 445 (25.9) | 28 (23‒32) |  | 99 (22.4) | 14 (10‒19) | 0.48 (0.38‒0.61) | <0.001 |
| >10 | 864 (50.3) | 35 (29‒41) |  | 148 (33.4) | 20 (15‒26) | 0.34 (0.28‒0.42) | <0.001 |
| **Outside Hubei** | 3200 (96.4) | 20 (16‒25) |  | 118 (3.6) | 17 (10‒25) |  |  |
| Age, years |  |  |  |  |  |  |  |
| <50 | 2016 (63) | 20 (16‒24) |  | 5 (4.2) | 18 (18‒30) | Reference |  |
| 50‒60 | 695 (21.7) | 21 (17‒27) |  | 12 (10.2) | 17 (12‒32) | 6.79 (2.4‒19.2) | <0.001 |
| 60‒70 | 325 (10.2) | 22 (18‒27) |  | 30 (25.4) | 19 (10‒30) | 33.56 (13.15‒85.65) | <0.001 |
| ≥70 | 164 (5.1) | 23(18‒28) |  | 71 (60.2) | 16 (8‒24) | 120.77 (48.79‒298.95) | <0.001 |
| Sex |  |  |  |  |  |  |  |
| Male | 1710 (53.4) | 20 (16‒26) |  | 74 (62.7) | 18 (12‒30) | 1.64 (1.13‒2.39) | 0.010 |
| Female | 1490 (46.6) | 20 (16‒25) |  | 44 (37.3) | 16 (9‒21) | Reference |  |
| Interval from symptom onset to admission, days |  |  |  |  |  |  |  |
| 1‒5 | 2751 (86) | 20 (16‒25) |  | 59 (50.0) | 12 (6‒20) | Reference |  |
| 6‒10 | 317 (9.9) | 23 (20‒26) |  | 38 (32.2) | 18 (13‒30) | 3.61 (2.38‒5.48) | <0.001 |
| >10 | 132 (4.1) | 27 (23‒31) |  | 21 (17.8) | 22 (16‒27) | 3.25 (1.97‒5.36) | <0.001 |

SHR, Sub-distribution hazard ratio; CI, confidence interval; IQR, interquartile range; COVID-19, coronavirus disease 2019.

*The results come from the multivariate analysis with age, sex, and the interval from symptom onset to admission.**Table S6. Clinical course of fatal cases and the factors associated with death among confirmed patients with SARS-COV-2 infection**

| **Characteristic** | Clinical course group | | |  | Univariate | |  | Multivariate | |
| --- | --- | --- | --- | --- | --- | --- | --- | --- | --- |
|  | 1‒10 | 11‒20 | >20 |  | OR (95% CI) | P |  | OR (95% CI) | P |
| **Age, years** |  |  |  |  |  |  |  |  |  |
| <50 | 5 (2.8) | 15 (7) | 3 (1.8) |  | Reference |  |  | Reference |  |
| 50‒ | 17 (9.4) | 21 (9.8) | 23 (13.9) |  | 1.5 (0.65‒3.46) | 0.337 |  | 1.19 (0.5‒2.83) | 0.685 |
| 60‒ | 50 (27.8) | 45 (20.9) | 49 (29.5) |  | 1.17 (0.55‒2.48) | 0.69 |  | 0.77 (0.35‒1.68) | 0.507 |
| ≥70 | 108 (60.0) | 134 (62.3) | 91 (54.8) |  | 1.05 (0.51‒2.16) | 0.885 |  | 0.8 (0.38‒1.68) | 0.553 |
| **Sex** |  |  |  |  |  |  |  |  |  |
| Female | 76 (42.2) | 74 (34.4) | 64 (38.6) |  | Reference |  |  | Reference |  |
| Male | 104 (57.8) | 141 (65.6) | 102 (61.5) |  | 1.13 (0.82‒1.55) | 0.449 |  | 1.08 (0.77‒1.49) | 0.661 |
| **Severity of disease** |  |  |  |  |  |  |  |  |  |
| Mild | 71 (39.4) | 84 (39.1) | 67 (40.4) |  | Reference |  |  | Reference |  |
| Severe | 109 (60.6) | 131 (60.9) | 99 (59.6) |  | 0.97 (0.71‒1.33) | 0.867 |  | 0.63 (0.44‒0.9) | 0.011 |
| **Interval from symptom onset to admission, days** |  |  |  |  |  |  |  |  |  |
| 1‒5 | 137 (76.1) | 67 (31.2) | 51 (30.7) |  | Reference |  |  | Reference |  |
| 6‒10 | 33 (18.3) | 66 (30.7) | 38 (22.9) |  | 2.76 (1.86‒4.11) | <0.001 |  | 2.84 (1.90‒4.24) | <0.001 |
| >10 | 10 (5.6) | 82 (38.1) | 77 (46.4) |  | 6.41 (4.34‒9.47) | <0.001 |  | 7.80 (5.19‒11.74) | <0.001 |
| **Location when diagnosed** |  |  |  |  |  |  |  |  |  |
| Outside Hubei | 32 (17.8) | 43 (20) | 43 (25.9) |  | Reference |  |  | Reference |  |
| Wuhan | 148 (82.2) | 172 (80) | 123 (74.1) |  | 0.7 (0.48‒1.02) | 0.065 |  | 0.42 (0.27‒0.65) | <0.001 |

**Table S7. Comparison on the characteristics of fatal cases with SARS-COV-2 infection between severe and mild cases as of 29 March 2020**

| **Characteristic** | **Severe**  **(n=339)** | **Mild**  **(n=222)** | **P value** |
| --- | --- | --- | --- |
| **Age, years, median (IQR)** | 72 (64‒80) | 75 (65‒82) | 0.131 |
| ≤10 | 0 (0) | 0 (0) | 0.445 |
| 10‒ | 0 (0) | 0 (0) |  |
| 20‒ | 1 (0.29) | 0 (0) |  |
| 30‒ | 8 (2.36) | 2 (0.9) |  |
| 40‒ | 7 (2.06) | 5 (2.25) |  |
| 50‒ | 36 (10.62) | 25 (11.26) |  |
| 60‒ | 92 (27.14) | 52 (23.42) |  |
| 70‒ | 101 (29.79) | 60 (27.0) |  |
| 80‒ | 94 (27.73) | 78 (35.1) |  |
| **Sex, n (%)** |  |  | 0.405 |
| Male | 205 (60.47) | 142 (64.00) |  |
| Female | 134 (39.53) | 80 (36.00) |  |
| **Interval from symptom onset to diagnosis, days, median (IQR)** | 8 (3‒13) | 8 (4‒14) | 0.742 |
| 1‒5 | 98 (28.91) | 70 (31.53) | 0.148 |
| 6‒10 | 105 (30.97) | 52 (23.42) |  |
| >10 | 136 (40.12) | 100 (45.05) |  |
| **Interval from symptom onset to admission, days, median (IQR)** | 7 (2‒11) | 5 (2‒10) | 0.118 |
| 1‒5 | 142 (41.89) | 113 (50.90) | 0.111 |
| 6‒10 | 88 (25.96) | 49 (22.07) |  |
| >10 | 109 (32.15) | 60 (27.03) |  |
| **Location of cases** |  |  | <0.001 |
| Wuhan | 227 (66.96) | 216 (97.30) |  |
| Outside Hubei | 112 (33.04) | 6 (2.70) |  |
| **Clinical course, median (IQR)** | 15 (9‒22) | 15 (9‒23) | 0.711 |

IQR, interquartile range.
